# The Tehran longitudinal family-based cardiometabolic cohort study sheds new light on dyslipidemia transmission patterns

**DOI:** 10.1038/s41598-024-53504-3

**Published:** 2024-02-27

**Authors:** Mahdi Akbarzadeh, Parisa Riahi, Amir Hossein Saeidian, Maryam Zarkesh, Sajedeh Masjoudi, Sara Asgarian, Kamran Guity, Hamed Moheimani, Homayoon Masoudi, Mahmoud Amiri Roudbar, Davood Khalili, Farhad Hosseinpanah, Maryam Barzin, Carolyn T. Hogan, Hakon Hakonarson, Mehdi Hedayati, Maryam S. Daneshpour, Fereidoun Azizi

**Affiliations:** 1 Cellular and Molecular Endocrine Research Center, Research Institute for Endocrine Sciences, Shahid Beheshti University of Medical Sciences, Tehran, Iran; 2 Center for Applied Genomics (CAG), Children’s Hospital of Philadelphia, 3615 Civic Center Blvd, Abramson Building, Philadelphia, PA 19104 USA; 3 Department of Surgery, University of Pittsburgh, Pittsburgh, PA USA; 4 Department of Animal Science, Safiabad-Dezful Agricultural and Natural Resources Research and Education Center, Agricultural Research, Education & Extension Organization (AREEO), Dezful, Iran; 5 Prevention of Metabolic Disorders Research Center, Research Institute for Endocrine Sciences, Shahid Beheshti University of Medical Sciences, Tehran, Iran; 6 Obesity Research Center, Research Institute for Endocrine Sciences, Shahid Beheshti University of Medical Sciences, Tehran, Iran; 7 Division of Hepatology, Temple University Hospital, Philadelphia, PA USA; 8 Department of Pediatrics, The Perelman School of Medicine, University of Pennsylvania, Philadelphia, PA 19104 USA; 9 Division of Human Genetics, Children’s Hospital of Philadelphia, Philadelphia, PA 19104 USA; 10 Division of Pulmonary Medicine, Children’s Hospital of Philadelphia, Philadelphia, PA 19104 USA; 11 Faculty of Medicine, University of Iceland, Reykjavik, Iceland; 12 Endocrine Research Center, Research Institute for Endocrine Sciences, Shahid Beheshti University of Medical Sciences, Tehran, Iran

**Keywords:** Computational biology and bioinformatics, Medical research

## Abstract

Dyslipidemia, as a metabolic risk factor, with the strongest and most heritable independent cause of cardiovascular diseases worldwide. We investigated the familial transmission patterns of dyslipidemia through a longitudinal family-based cohort, the Tehran Cardiometabolic Genetic Study (TCGS) in Iran. We enrolled 18,729 individuals (45% were males) aged > 18 years (mean: 38.15 (15.82)) and observed them over five 3-year follow-up periods. We evaluated the serum concentrations of total cholesterol, triglyceride, high-density lipoprotein cholesterol, and low-density lipoprotein cholesterol with the first measurement among longitudinal measures and the average measurements (AM) of the five periods. Heritability analysis was conducted using a mixed-effect framework with likelihood-based and Bayesian approaches. The periodic prevalence and heritability of dyslipidemia were estimated to be 65.7 and 42%, respectively. The likelihood of an individual having at least one dyslipidemic parent reveals an OR = 6.94 (CI 5.28–9.30) compared to those who do not have dyslipidemic parents. The most considerable intraclass correlation of family members was for the same-sex siblings, with ICC ~ 25.5%. For serum concentrations, heritability ranged from 33.64 to 60.95%. Taken together, these findings demonstrate that familial transmission of dyslipidemia in the Tehran population is strong, especially within the same-gender siblings. According to previous reports, the heritability of dyslipidemia in this population is considerably higher than the global average.

## Introduction

Dyslipidemia, as a metabolic risk factor, is the strongest and most heritable independent cause of cardiovascular diseases (CVD)^1^, comprising nearly 50% of all non-communicable diseases, including hypertension and type 2 diabetes (T2D). The prevalence of hypercholesterolemia and hypertriglyceridemia in Iranian adults is 27.1 and 19.4%, respectively^2^. Despite a high incidence of high-risk lipid profiles in the Tehran Lipid and Glucose Study (TLGS) population, favorable trends in lipid component levels were detected recently^3,4^.

Dyslipidemia in the Iranian population has increased due to various causes, including geographic circumstances, climatic change, socioeconomic status, and significant lifestyle and diet changes^5^. Nevertheless, these well-established risk factors share a familial resemblance, the degree of similarity between each family member, which may be assessed through correlations or covariances among family members^6^. Examining familial resemblance between different types of relative pairs sheds light on the shared genetic basis of lipid profile traits. Numerous studies have been conducted globally to determine the family resemblance and correlation of lipid characteristics as components of metabolic syndrome^7–13^. Also, positive assortative mating, shared environmental circumstances, cohabitation influences, age effects, or any combination of these variables may contribute to spousal similarities. Even in couples who are not biologically related, their obesity, lipid profile, and overall health are more similar than in a random sampling of individuals^14^.

Heritability measures the degree of familial resemblance and is expressed as the proportion of variation explained by all additive familial effects, including genetic and familial shared environment effects^6^. Dyslipidemia is a highly heritable disease, and research has been undertaken in Asian, European, and American populations to determine the heritability of lipid levels in families worldwide^9–11,15–19^. Recently, in the Iranian population, family-based heritability for low HDL-C and high TG has been determined to be 40 and 34%, respectively^20,21^.

Through a longitudinal family-based cohort study, the Tehran Cardiometabolic Genetic Study (TCGS) represents multidimensional data to evaluate transmission patterns of dyslipidemia among families. In this study, we aimed to determine a new definition of dyslipidemia in TCGS based on longitudinal lipid profile measures and to investigate the family history of dyslipidemia, periodic prevalence of dyslipidemia, familial correlation, family-based heritability (h^2^_family_), and spousal resemblance for the dyslipidemia status and each lipid profile trait in TCGS participants. Familial resemblance and heritability were estimated using two scenarios: (i) the first measurement (FM) among longitudinal assessments and (ii) the average of the measurements (AM). In addition, we aimed to study the discrepancy in the family resemblance between the two scenarios.

## Materials and methods

### Study participants and genealogy data

Subjects and their families were selected from TCGS^22,23^, an ongoing cohort study running in the Tehran Lipid and Glucose Study (TLGS). In the TLGS, 20,276 participants from District 13 of Tehran were followed for important cardiovascular and metabolic health events, such as obesity and dyslipidemia, throughout the past 23 years. Six follow-up periods were conducted, with a roughly three-year interval between two consecutive phases with 20,276 participants (started in 1999). The study's full scope and methodology are described elsewhere^24^. Under the framework of the TLGS, the Tehran Cardiometabolic Genetic Study (TCGS) has been conducted as a family-based longitudinal framework study to identify potential targets for the prevention and intervention of non-communicable diseases developing in mid-life and late life, focusing on cardiovascular, endocrine, metabolic abnormalities, cancers, and some inherited diseases^22,23^. The study encompasses a diverse range of demographic characteristics, including different ethnic groups, and employs a variety of methods, including genotyping and health-related complication encoding. The findings contribute significantly to understanding cardiometabolic disease in the Iranian population and implementing precision medicine.

This study selected participants from the last five TLGS/TCGS phases from 2002 to 2017 to provide highly validated lipid serum level measurements. There are 3,102 family members (min = 3; max = 56), with 18,729 participants chosen following the TCGS's objectives, of which 1,766 are self-identified independents.

**Figure 1:**
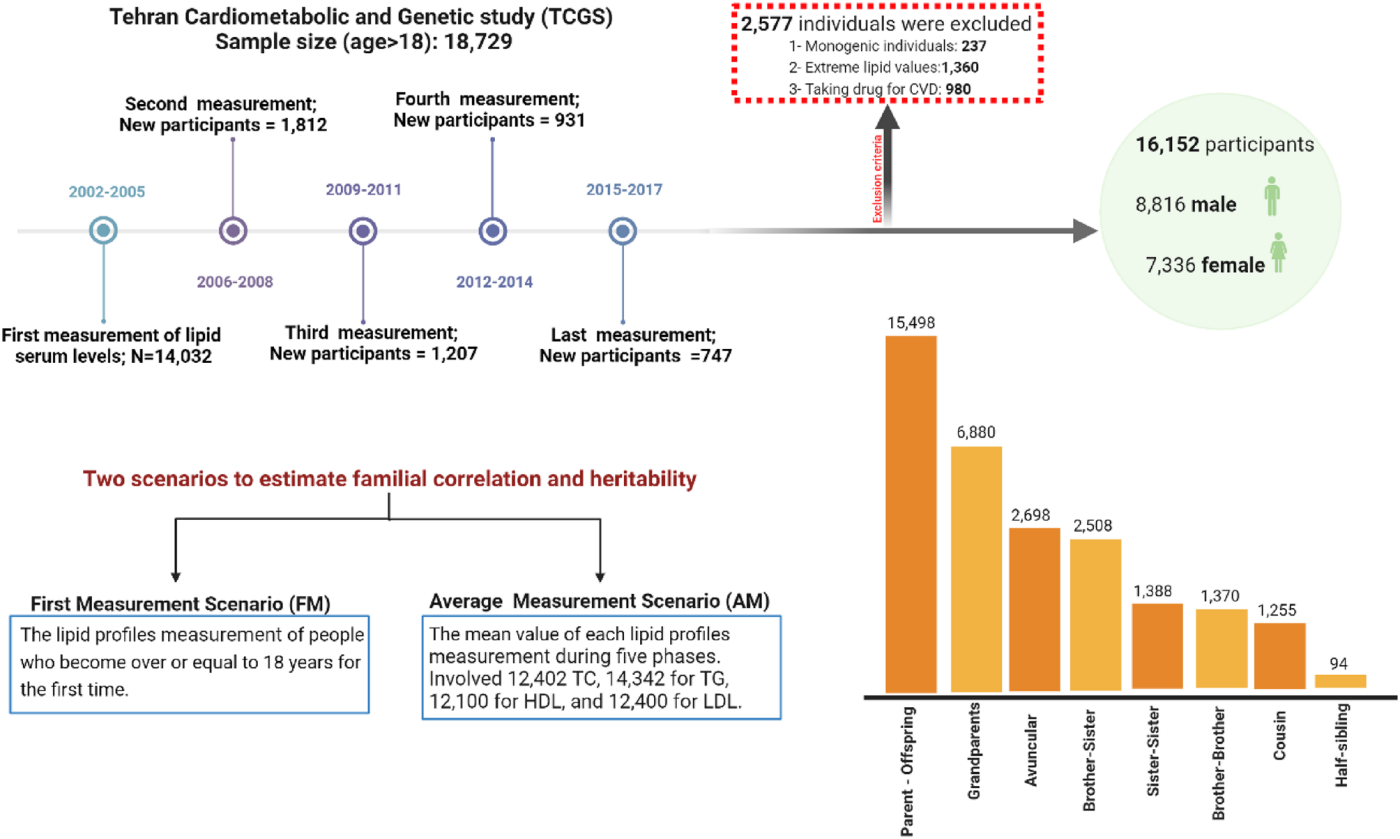
In each of the 5 phases of the TCGS study, individuals older than 18 years were selected. With this inclusion criterion, in the first phase, 14,032 people were selected, and in subsequent phases, respectively 1,812, 1,207, 931, and 747 people were chosen for measuring lipid profiles. Based on the exclusion criteria, 2,577 people were excluded from the study, and ultimately, 16,152 people (8,816 men, (54%)) entered the study. The distribution of relative pairs also shows that the number of parent–offspring pairs is more than other types.

Figure 1 illustrates the study's design, showing the initial selection of 14,032 individuals from 2002 to 2005. It also details the addition of new participants in four subsequent phases: 1,812 in the first, 1,207 in the second, 931 in the third, and 747 in the fourth phase.

### Inclusion and exclusion criteria

Inclusion criteria:

Individuals older than or equal to 18 years of age.

Exclusion criteria:

1. People with extreme lipid serum levels were excluded.
2. Individuals with monogenic dyslipidemia diseases, such as Familial Hypercholesterolemia (FH) based on Simon Broome criteria, and their families with positively related histories^25–27^.
3. Participants who were < 40 years of age with a positive lipid-lowering drug history were excluded.
4. Individuals older than 40 years of age, taking lipid-lowering medication or having a positive cardiovascular status as determined by the TLGS hospitalization outcome questionnaire.

Ultimately, 16,152 individuals were recruited for the study, as shown in greater detail in Fig. 1.

### Collecting genealogy data, drawing pedigrees, and relationship testing

From 2012 to 2017, all participants' genealogical information was gathered, including their kinship relationship to the homeowners and marital status (Consanguineous marriage rate: 28.15%). When participants' relationship status changed over time, they were interviewed to note it in genealogy data. Guidelines for standardizing human pedigree nomenclature were used to establish biological ties^31,32^. Each person was assigned a unique identification number (ID), and spouse pairs were identified by having descendants or spouses who did not have children. The Statistical Analysis for Genetic Epidemiology (SAGE) software was used to check family member relationships^33^. Kinship relations and accuracy of drawing pedigrees were also controlled by the Family-Based Association Tests (FBAT-Toolkit V 1.7.3)^34^. There were no more missing data in any clusters since all adoption cases, family separations, remarriages, and name changes had been thoroughly rechecked. The genetic data management system Progeny Clinical Version 7 saved and processed family data, pedigree information, phenotypic data, and genotype data (Progeny Software LLC, Delray Beach, FL)^35^.

### Ethics declaration and consent form

The local research ethics committee of the Iran National Science Foundation approved this study (Research Approval Code 95014470 and Research Ethical Code: IR.SBMU.ENDOCRINE.REC.1398.135). All participants provided written informed consent for participating in the study. This research was conducted in compliance with the Helsinki Declaration.

### Laboratory measurements

After 12 h of fasting, venous blood samples were collected for biochemical analysis. Total Cholesterol (TC) was measured using the enzymatic colorimetric method with cholesterol esterase and oxidase. Triglycerides (TG) were measured using glycerol phosphate oxidase. High-density lipoprotein cholesterol (HDL-C) was computed after precipitation of the apolipoprotein B containing lipoproteins with phosphotungstic acid. In all assays, inter- and intra-assay CVs were < 1.9, 2.1, and 3% for TC, TG, and HDL-C, respectively. The modified Friedewald formula based on TG, TC, and HDL-C was used to calculate low-density lipoprotein (LDL-C)^36^. Analyses were performed using Pars Azmon kits (ParsAzmon, Tehran, Iran) and a Selectra 2 auto-analyzer (Vital Scientific c, Spankeren, Netherlands). All samples were analyzed only when internal quality control met acceptable criteria. The levels of TC were adjusted by dividing 0.8 for those receiving the lipid-lowering drug^37^.

### Definitions

The thresholds used for lipid profile traits based on ATP III criteria were high TC (> = 200 mg/dL), high TG (> = 200 mg/dL), low HDL-C (< 40 mg/dL for men and < 50 mg/dL for women), and high LDL-C (> = 160 mg/dL)^38–41^. Individuals with dyslipidemia had a positive lipid-lowering drug history, were over the age of 40, or met at least one of the thresholds in at least two phases of TLGS.

### Statistical analysis

Lipid traits, TC, and log transformation of TG, HDL-C, and LDL-C in each TCGS phase were adjusted for age and gender. After that, values were transformed into the normal distribution through an inverse normal transformation using the RNOmni package in R software^42^.

We considered two scenarios, FM and AM, to estimate familial correlation and heritability. The first scenario considered the lipid profiles of people who become over or equal to 18 years old for the first time, named FM through the paper. To reduce measurement error through parameter estimation, we evaluated the second scenario based on average lipid profile traits, referred to as the AM (average measurement). In the AM scenario, the analysis involved 12,402 participants for Total Cholesterol (TC), 14,342 for Triglycerides (TG), 12,100 for High-Density Lipoprotein (HDL), and 12,400 for Low-Density Lipoprotein (LDL).

Participants were classified into "dyslipidemic" and "non-dyslipidemic" according to their lipid profile levels, age, lipid-lowering drug intake, and cardiovascular status during the TLGS cohort.

#### Periodic prevalence of dyslipidemia in TCGS

Periodic prevalence of a disease is regarded as the proportion of the population with the disease condition during the given period (e.g., TCGS phase) plus the point prevalence at the start^43^. This study estimated point prevalence at the second phase of TCGS (this study's first point), and the new cases were captured from the following phases, three to six.

#### Familial history assessment

Individuals were adjusted by age and sex in a logistic regression model to determine the influence of a family history of dyslipidemia (First-degree relatives) on an individual's risk of being afflicted in three settings (within males, females, and total). The significant level was considered at 0.05, and the models were also performed using R version 4.0.3. Pedigree information was obtained using the SAGE software^33^.

#### Familial aggregation and spousal resemblance of lipid profile traits

The intraclass correlation (ICC) coefficients of all relative pairs were estimated for both FM and AM using the FCOR command of the SAGE software to verify family resemblance for all relative pairs and spouses. Due to the vast number of relative-pair types, only significant ICCs were held.

#### Family-based heritability

Classical likelihood-based and Bayesian approaches were used to assess the family-based heritability of lipid profile traits. Using the former, it is possible to estimate polygenic heritability and additional family correlation parameters, which perform likelihood ratio tests and generate maximum likelihood estimates assuming multivariate normality following either George–Elstone or Box-Cox transformation^33^. In the latter, a kinship matrix from TCGS was used to estimate heritability as a random and fixed factor, including age and sex. A Gaussian random-effects model with a covariance structure was used. We included a random effect, $k=N(0, K{\sigma }_{g}^{2})$, where $K$ is a kinship matrix, and ${\sigma }_{g}^{2}$ is the genetic variance. The response vector $y={\{y}_{i}\}$ was defined as the lipid profile trait levels for the i^th^ individual. Non-Gaussian outcomes were accommodated using the probit link under a Bayesian Markov chain Monte Carlo (MCMC) setting. The probit link was implemented as $\left({K}_{i}\right) =\Phi \left({\eta }_{i}\right)$, where Φ is the cumulative distribution function (CDF) and ${\eta }_{i}$ is a linear predictor given by:

$${\eta }_{i}=\mu +{\sum }_{j=1}^{q}{x}_{ij}{\beta }_{ij}+{k}_{i}.$$

where $\mu$ is an intercept, ${x}_{ij}$ is the kth fixed factors, ${\beta }_{ij}$ is the effects associated with the kth fixed factors, and ${k}_{i}$ is a total genetic effect of the ith individual. Our Bayesian analysis was implemented using the BGLR R package^44^. The number of iterations of the Gibbs sampler was 400,000, where the first 200,000 samples were discarded as burn-in. A thinning interval of 40 was used. Thus, 5,000 posterior samples were used to compare the features of the posterior distribution. The convergence was visualized through trace plots of all the unknown values and computation of the Gelman-Rubin statistic for convergence below 1.03^45^.

## Results

### Demographic characteristics of participants

There were 18,729 eligible participants, all of whom were over the age of 18 (Fig. 1). The males and females were 8,484 (45.3%) and 10,245 (54.7%), with an average age of 38.24 (16.86) and 36.76 (15.43) years old, respectively. Information about pedigree and relative pairs is provided in Table 1. There were 3,102 families with a mean of 3.24 (2.91) members and a range of 3 to 56 members, with 1,766 singletons and 5,266 sibling relationships (1.70 (0.92); range: 1–8). There were 20,764 first-degree relatives (15,498 parents or children and 5,266 siblings) and 10,922 s-degree relatives, including grandparents, avuncular, half-siblings, and cousins. Table 2 provides the mean (SD) of lipid serum levels by gender for both scenarios (FM and AM). The mean difference for each variable was tested via the corrected two independent samples t-test. In both scenarios, these results revealed a significant difference in the mean values between men and women (p < 0.001).

**Table 1:** Relative-pairs information of the TCGS participants.

Pairs type		Number of pairs	
First-degree relative	Parents/offspring		15,498
	Siblings (5266 pairs)	Sister–sister	1388
		Brother–brother	1370
		Brother–sister	2508
Second-degree relative	Grandparents		6880
	Avuncular		2693
	Half sibling		94
	Cousin		1255

**Table 2:** Baseline characteristics of participants in terms of age and lipid serum levels.

Variable	N = 16,152 (males = 45.30%)	FM	P-value*	AM	P-value*
		Mean (SD)		Mean (SD)	
Age (years)	Males	38.24 (16.86)	< 0.0001	36.95 (17.6)	< 0.0001
	Females	36.76 (15.43)		35.01 (14.28)	
Total cholesterol (mg/dl)	Males	194.43 (45.6)	< 0.0001	192.56 (46.75)	< 0.0001
	Females	182.56 (80.3)		180.14 (82.59)	
Triglyceride (mg/dl)	Males	169.86 (122.97)	< 0.0001	167.23 (120.55)	< 0.0001
	Females	132.68 (109.5)		131.92 (111.08)	
HDL-C (mg/dl)	Males	38.8 (9.66)	< 0.0001	37.04 (11.37)	< 0.0001
	Females	40.11 (19.3)		38.57 (18.57)	
LDL-C (mg/dl)	Males	133.32(39.37)	< 0.0001	132.11(21.1)	< 0.0001
	Females	106.16 (57.24)		102.6(42.19)	

FM: First Measurement; AM: Average Measurements; SD: Standard Deviation.

*P-value obtained from corrected two independent samples t-test.

### Periodic prevalence of dyslipidemia

Point prevalence for the second phase of TCGS was estimated to be 40.57%, and by entering the new cases of dyslipidemia, we have estimated that the periodic prevalence of dyslipidemia was 65.75%.

### Familial aggregation of lipid traits and dyslipidemia

**Figure 2:**
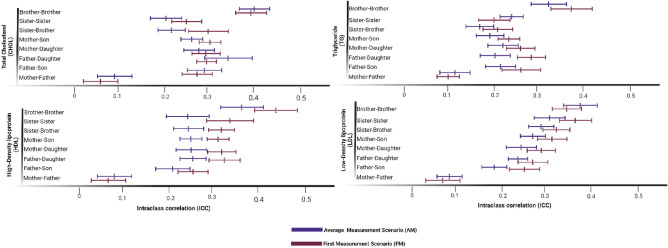
Significant pairwise correlation (ICC) for lipid serum levels between family members of TCGS; the pairwise correlation among the siblings revealed significant brother-brother correlations for all lipid traits based on two measurement scenarios. Generally, the siblings' correlations were higher for all lipid traits than others. Spousal resemblance among TCGS participants for all lipid serum levels was significant, with relatively lower ICC than in other first-degree relatives.

Based on two scenarios, all types of first-degree relative pairs and spouses among TLGS participants have been considered for the familial correlation of four lipid serum levels. We picked only those with a significant intraclass correlation (ICC) out of all relative pairs. Figure 2 depicts these significant ICCs with a 95% confidence interval of lipid serum levels among TLGS subjects.

For almost all of the phenotypes in the FM scenario, when the first-degree relative pairs are considered, brother-brother has the largest ICC among all pairs (LDL-C: 38.88%, HDL-C: 35.44%, TC: 40.49%, and TG: 30.96%), followed by sister-sister (LDL-C: 31.27%, HDL-C: 24.71%, TC: 19.23%, and TG: 22.62%). Although the estimated correlations among first-degree relative pairs in the AM scenario are smaller than those in the FM scenario, the strongest correlation is estimated for the brother-brother pair for all lipid traits.

However, the trend is the opposite in spousal correlations for lipid levels. Generally, the correlations estimated based on the FM scenario are more significant than the AM scenario. As for the FM scenario, ICC for LDL-C, HDL-C, TC, and TG were 9.30, 9.55, 9.75, and 10.82%, respectively, while for the AM scenario, it was 8.44, 5.57, 8.33, and 9.99%.

**Figure 3:**
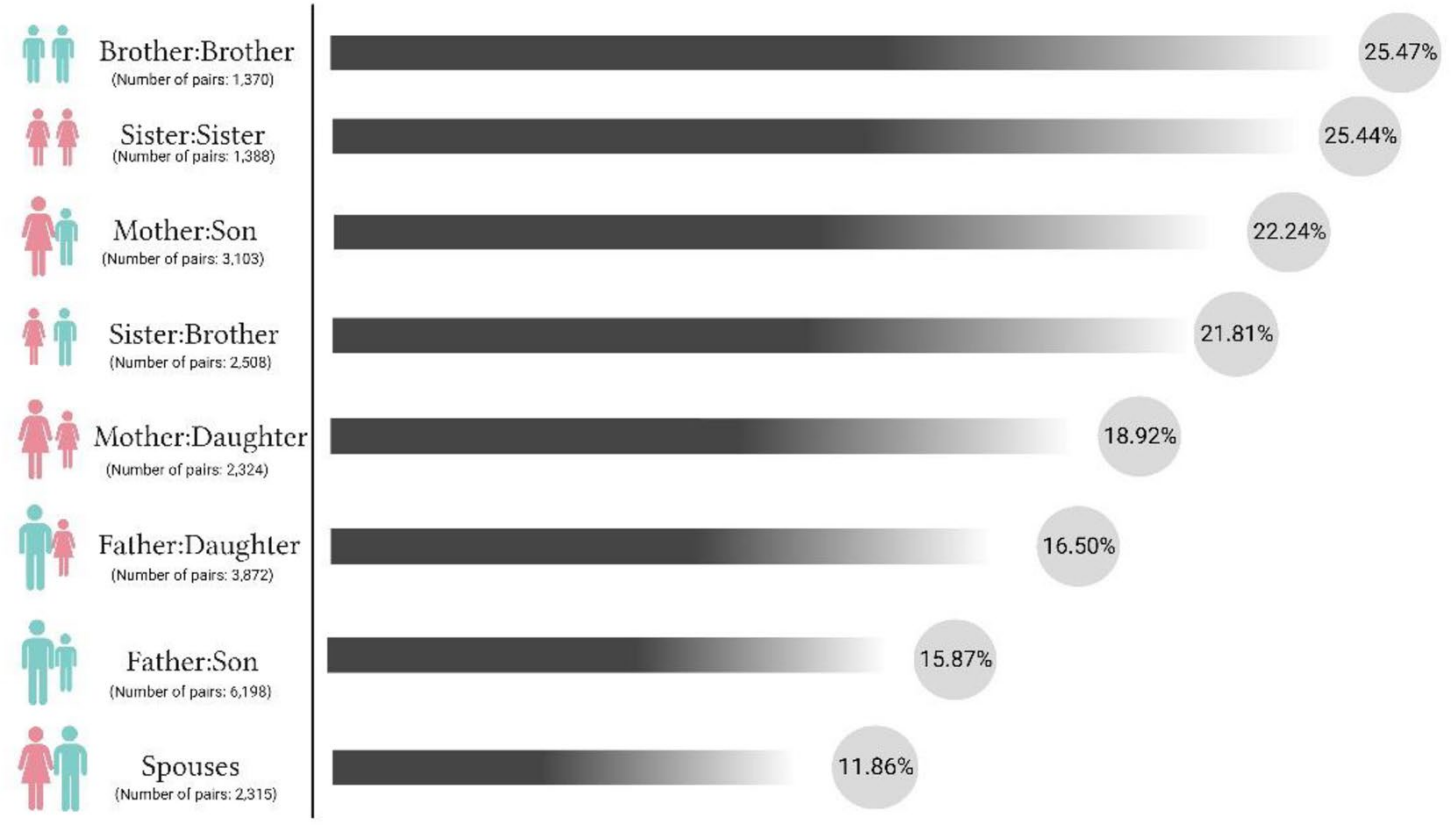
Pairwise correlation for dyslipidemia between family members of TCGS; taking into account the familial patterns in dyslipidemia, the strongest correlation among family members is observed between siblings of the same sex. In the case of parent–offspring relationships, the highest ICC is noted between mothers and their sons.

Considering the familial resemblance for dyslipidemia, the most considerable correlation between family members belongs to siblings of the same gender, with an ICC of ~ 25.4%. Regarding parents and offspring, the largest ICC belongs to mothers and their sons, with an ICC of 22.42%, and their daughters, with an ICC of 18.92%, as the second largest ICC (Fig. 3).

**Figure 4:**
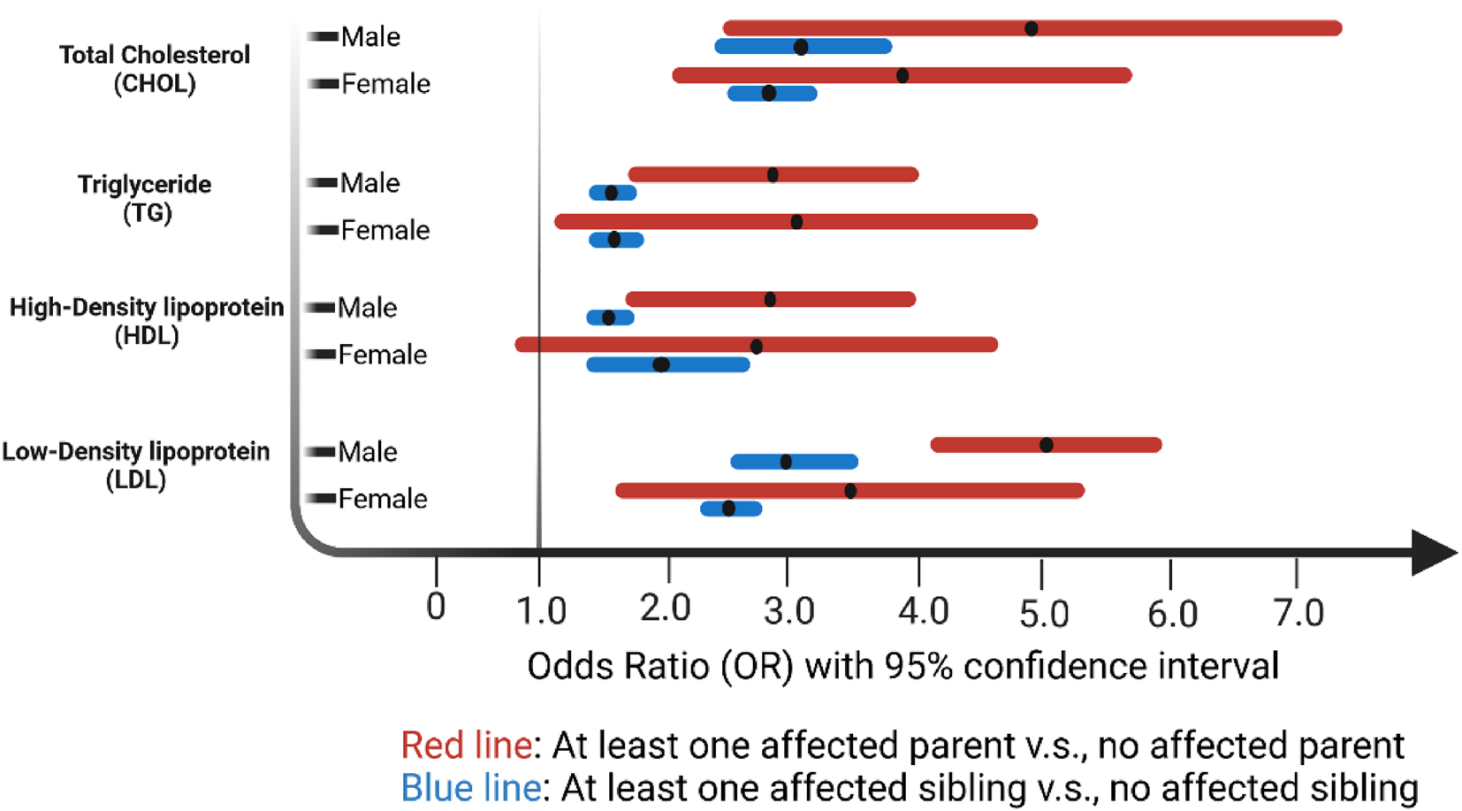
The effect of a positive family history of dyslipidemia among TCGS individuals, specifically focusing on parents with dyslipidemia, reveals that individuals, regardless of gender, who have at least one parent with dyslipidemia are significantly more likely to inherit dyslipidemia compared to those without any dyslipidemic parents.

Analysis of the positive familial history of dyslipidemia considering the dyslipidemic parents (Fig. 4) shows the chance of an individual, either female or male, with at least one dyslipidemic parent inheriting dyslipidemia is significantly higher than those with no dyslipidemic parent. This trend is evident in almost all four lipid traits, as the odds of developing dyslipidemia for those with at least one dyslipidemic parent against those with healthy parents is OR_TC_ = 3.67 (CI: 2.49–5.59), OR_TG_ = 2.52 (CI: 1.71–3.73), OR_HDL-C_ = 2.04 (CI: 1.46–2.86), and OR_LDL-C_ = 3.97 (CI: 2.32–5.62). Also, results show that the odds of individuals with at least one dyslipidemic parent against those with healthy parents with dyslipidemia amounts to OR = 6.94 (CI: 5.28–9.30). The odds ratios are fully presented in Supplemental File 1.

### Family-based heritability

Table 3 provides the family-based heritability of lipid serum levels based on two scenarios (FM and AM) using classical likelihood-based and Bayesian approaches (BGLR). The results of the likelihood-based method depict that in the AM scenario, the family-based heritability of lipid serum levels ranges between 48.31% for TG and 60.95% for HDL-C. In the FM scenario, the variances explained by the pedigree range between 33.64% for TG and 42.58% for TC. The Bayesian (BGLR) approach shows that the family-based heritability of lipid serum levels ranges from 34.60 to 34.70% for FM and from 34.60 to 34.80% for AM. The likelihood-based method consistently produced higher AM heritability than FM. However, heritability resulting from the Bayesian (BGLR) approach for AM and FM is very close. Family-based heritability of dyslipidemia is estimated to be 41.07% (SE: 0.023).

**Table 3:** Family-based heritability of Lipid serum level of TCGS participants.

Lipid serum level	LMM Methods	FM	AM
		Heritability (%) (SE)	Heritability (%) (SE)
Total Cholesterol (mg/dl)	Likelihood-based	42.58 (4.21 × 10^–4^)	52.56 (3.43 × 10^–4^)
	Bayesian	34.80 (0.00265)	34.80 (0.00262)
Triglyceride (mg/dl)	Likelihood-based	33.64 (4.29 × 10^–4^)	48.31 (3.55 × 10^–4^)
	Bayesian	34.70 (0.00265)	34.60 (0.00275)
HDL-C (mg/dl)	Likelihood-based	40.50 (4.25 × 10^–4^)	60.95 (3.10 × 10^–4^)
	Bayesian	34.70 (0.00276)	34.80 (0.00272)
LDL-C (mg/dl)	Likelihood-based	41.10 (4.2 × 10^–4^)	51.57 (3.46 × 10^–4^)
	Bayesian	34.60 (0.00265)	34.70 (0.00268)

FM: First Measurement; AM: Average Measurements; SE: Standard Error; LMM: Linear Mixed Model.

## Discussion

The current study presents a ground-breaking investigation where various methodologies were employed to determine the periodic prevalence, familial aggregation, spousal similarity, and family-based heritability of lipid profile traits in the Iranian population.

Analysis of the familial transmission revealed that for almost all lipid traits, the likelihood of an individual, either female or male, with two dyslipidemic parents inheriting dyslipidemia is significantly higher than in those with a healthy parent. The pairwise correlation among the siblings revealed significant brother-brother correlations for all lipid traits based on two measurement scenarios. Generally, the siblings' correlations were higher for all lipid traits than others. Spousal resemblance among TCGS participants for all lipid serum levels was significant, with relatively lower ICC than in other first-degree relatives.

The primary reason for investigating familial aggregation and transmission of lipid serum levels is to validate the recent research findings from other nations regarding the high aggregation of lipid serum level traits in Iran. We know about the heredity of lipid traits based mainly on prior studies that investigated the variance that accounts for lipid serum levels worldwide. As a result, there is an urgent need to fill the gap of unknown heritability of serum levels in Iran and determine whether this familial aggregation is caused by high heritability in families. The pattern of significant correlation between siblings shows that familial aggregation is partly related to hereditary factors^46^. The findings of this study confirmed the familial aggregation and transmission of lipid serum levels among the Iranian population, which is similarly reported in several recent studies globally in other populations^47–52^. The family-based heritability of lipid serum levels is the other crucial feature of the study. Using the kinship matrix, we have investigated the phenotypic variance of lipid traits due to pedigree and familial relationships^21^.

Globally speaking, the recent decades have beheld a series of studies on the heredity of lipid profiles^7,9–15,45^. According to these studies, family-based heritability of LDL-C ranged between 0.32 and 0.69. Considering HDL-C, the variance explained by pedigree ranged between 0.23 and 0.8. Coming to TC, the heritability related to familial information was reported to be between 0.42 to 0.67. However, these values differed between 0.17 and 0.68 for TG family-based heritability. Considering that heritability differs among populations and relies on the inclusion criteria, disease, treatment effects, medication, and statistical model factors, the findings of this study confirmed the recent research on the heredity of lipid serum levels, as they show that lipid traits are moderate to highly heritable^53^.

Spousal pairs permit the assessment of determinants of diseases related to the environment because they share the same lifestyle and environment. Recent studies have reviewed spouses' concordance with the major coronary risk factors and have reported significant correlations among spouses for lipid serum levels. According to a meta-analysis, 10–15 studies reported a significant but minor spousal resemblance for TG, TC, and LDL-C, but not HDL-C. However, others have suggested that the HDL-C correlation between spouses increased with age, which would be regarded as due to cohabitation. These findings verified published reports on the spousal resemblance of lipid serum levels^54^.

The Iranian people are divided into a cluster of similar and mixed groups and have had several language adoption events during bygone eras. It has been previously proven that Iranians exhibit a distinctive genetic variation while having proximity to surrounding populations. These variations are consistent with long-term genetic continuity and harbor significant heterogeneity and varying levels of consanguinity. Due to the differences between the ancestry of Iranian ethnicities and other nations, the results of this study are vital to address the heterogeneity between the Iranian population and others for checking the heredity of lipid profile traits. Additionally, the findings of this research are likely to be helpful for further research on lipid traits in Iran. More importantly, this study unveiled the intuition and evidence needed to start profound genetic studies on lipid traits^55^.

Considering all of the strengths mentioned earlier in this study, it could be improved in several ways. Comparing the sample size of TLGS/TCGS with the other current cohorts in the world, we have a relatively small sample size. The power of a study to detect meaningful differences or associations increases with the number of participants. Expanding the cohort size could involve recruiting more participants from a broader demographic within Tehran. Additionally, longitudinal studies with follow-ups could provide more comprehensive data over time. The study focuses on Tehranian families, which might limit the generalizability of its findings to other populations. While it's valuable to have a representative sample of Tehranian families, comparing these findings with data from other cohorts in diverse geographical and cultural settings can enhance the understanding of how the results apply to broader populations. Collaborations with international cohorts could also be explored.

Another limitation could be the need for more genomic-based results, which could be differentiated in terms of shared environment and genetic effects. Integrating more genomic analyses can provide insights into the genetic basis of cardio-metabolic diseases. This could involve genome-wide association studies (GWAS), whole-exome, or whole-genome sequencing. Understanding the interplay between genetics and environmental factors is crucial for a comprehensive understanding of these diseases. However, there is a need to distinguish between the influences of shared environments and genetics on the study outcomes. Employing statistical models that can separate these effects is essential. This could include twin studies, sibling designs, or the use of advanced statistical techniques like structural equation modeling. Overall, while the TCGS provides valuable insights, these improvements could significantly enhance the scope and impact of the study, leading to a more comprehensive understanding of cardio-metabolic diseases in the Iranian population and beyond. As a following study, we suggested designing and conducting a genomic-based study for distinguishing shared environmental and additive genetic effects of lipid-related traits within TCGS participants.

## Conclusion

Taken together, our findings establish that familial transmission of dyslipidemia in the Tehran population is highly robust, especially within same-gender siblings. According to previous reports on heritability, the shared-environmental proportion attributed to the variation of dyslipidemia in this population is considerably higher than the global average.

Scientists have a consensus that heritability captured by pedigree information is further enhanced by the effect of non-genetic factors, like shared environment^56^. Hence, this issue may motivate scientists to estimate the genomic-based heritability of lipid traits among the Iranian population to exclude the effect of the shared environment.

### Supplementary Information

Supplementary Information.

## Data Availability

The datasets generated and/or analyzed during the current study are not publicly available due to the privacy of research participants but are available from the corresponding author on reasonable request.
